# Adaptive Second-Order Fixed-Time Sliding Mode Controller with a Disturbance Observer for Electronic Throttle Valves

**DOI:** 10.3390/s23187676

**Published:** 2023-09-05

**Authors:** Yinkai Feng, Yun Long, Chong Yao, Enzhe Song

**Affiliations:** 1 Yantai Research Institute, Harbin Engineering University, Yantai 264000, China; fyk@hrbeu.edu.cn (Y.F.);; 2 College of Power and Energy Engineering, Harbin Engineering University, Harbin 150001, China

**Keywords:** adaptive sliding mode control, electronic throttle, fixed-time convergence, second-order sliding mode control, disturbance observer

## Abstract

In order to enhance the precision and speed of control for electronic throttle valves (ETVs) in the face of disturbance and parameter uncertainties, an adaptive second-order fixed-time sliding mode (ASOFxTSM) controller is developed, along with disturbance observer compensation techniques. Initially, a control-oriented model specifically considering lumped disturbances within the ETV is established. Secondly, to address the contradiction between fast response and heavy chattering of conventional fixed-time sliding mode, a hierarchical sliding surface approach is introduced. This approach proficiently alleviates chattering effects while preserving the fixed convergence properties of the controller. Furthermore, to enhance the anti-disturbance performance of the ETV control system, an innovative fixed-time sliding mode observer is incorporated to estimate lumped disturbances and apply them as a feed-forward compensation term to the ASOFxTSM controller output. Building upon this, a parameter adaptive mechanism is introduced to optimize control gains. Subsequently, a rigorous stability proof is conducted, accompanied by the derivation of the expression for system convergence time. Finally, a comparison is drawn between the proposed controller and fixed-time sliding mode and super-twisting controllers through simulations and experiments. The results demonstrate the superiority of the proposed method in terms of chattering suppression, rapid dynamic response, and disturbance rejection capability.

## 1. Introduction

The electronic throttle valve (ETV) serves as the primary actuator for controlling the air intake of an engine, directly influencing the power and fuel efficiency of the engine. Traditional throttle control mechanisms, such as throttle by wire (TbW), often suffer from wear and failure [1]. To overcome these challenges, the adoption of electronic throttle valve technology has gained prominence. ETVS offer enhanced reliability, stability, and reduced maintenance costs [2,3,4]. However, controlling the ETV is complex due to various nonlinear factors, including stick-slip friction, gear clearance, and discontinuous nonlinear springs [5]. In recent years, several control strategies have been proposed for ETV, including PID control [6,7,8], optimal control [9,10], adaptive control [11,12,13,14], and sliding mode control [15,16,17,18]. Among them, sliding mode (SM) control is a powerful nonlinear control method that can achieve stable and robust control even in the presence of model uncertainties and external disturbances, which makes SM well-suited for ETVs.

Initially, the plinear sliding mode (LSM) was predominantly used in ETVs. For instance, Song et al. [19] and Humaidi et al. [20] proposed LSM controllers for automotive electronic throttle using the backstepping method. However, the LSM’s sliding surface can only guarantee asymptotic convergence, limiting the performance of SM in ETVs. To address this limitation, researchers have explored the terminal sliding mode (TSM), which achieves finite-time tracking of the throttle valve target value. Wang et al. [21] adopted the TSM with a nonlinear sliding surface, and Song et al. [22] further improved the approach and applied the fast terminal sliding mode (FTSM) to ETVs. While these methods provide quicker dynamic responses and greater accuracy in position tracking, the settling time still relies on the initial state of the ETV, posing a challenge in estimating and ensuring an upper bound for the settling time.

To resolve the problem of convergence time being affected by the initial state of the system, Polyakov [23] proposed the theory of fixed-time stability, where the settling time is solely determined by the controller’s parameters, independent of the system’s initial state. This theory has significant implications for enhancing system dynamic performance by accelerating convergence speed and introducing a reference index. Building on this theory, Li et al. [23] applied fixed-time stability to propose a fixed-time non-singular terminal sliding mode, and Huang et al. [24] developed a fixed-time fractional-order sliding mode controller for the wind turbines. Hu et al. [15] proposed a fixed-time sliding mode adaptive trajectory controller for ETVS based on extreme learning machines.

It is worth noting that fixed-time stability theory does not explicitly address the chattering problem inherent in SM. Conversely, in some systems, fixed-time controllers can cause more serious chattering problems than conventional controllers [25]. Therefore, further research is necessary to suppress chattering while ensuring fixed-time convergence. Researchers have explored various approaches to mitigate chattering, such as the concept of Quasi-Sliding Mode (QSM) introduced by Slotine et al. [26]. QSM reduces chattering by adopting a relatively smooth switching function. Building upon this concept, Trujillo et al. [27] and Ma et al. [28] proposed more advanced solutions. However, it is crucial that reducing the impact of the switching function might undermine the controller’s robustness, making it unsuitable for the ETV system with numerous disturbances. In recent years, several advanced control methods have emerged, including neural networks and adaptive control. These methods are also considered effective approaches to address the chattering issue in sliding mode control. The principle of these methods is to reduce the required switching control gain by compensating for the disturbance and uncertainty. Feng et al. [29] introduced a novel adaptive sliding mode control method based on RBF neural networks (SMC-RBF), utilizing RBF neural networks to compensate for model uncertainty and disturbance. Narayan et al. [30]. proposed a robust adaptive backstepping control to deal with model uncertainties and external disturbances of a lower-limb exoskeleton system. Similarly, Ma et al. [31] proposed an adaptive backstepping sliding mode fault-tolerant controller and effectively solved the chattering problem in the control of the wind turbine system. Further, Liu et at. [14] and Wang et al. [13] applied adaptive control to the electronic throttle system and made progress in the tracking error. However, in specific applications, ensuring the stability of these intelligent methods is challenging, and incorrect parameter update rules could result in system instability. This challenge is particularly pronounced when addressing the chattering problem in fixed-time sliding mode, as striking a balance between rapid convergence and stability proves intricate.

Furthermore, another effective method to address chattering is high-order sliding mode (HOSM) control. Levant [32] first introduced the concept of HOSM in 1993. Researchers such as Lochan et al. [33], Zhou [34], Wang et al. [35], and Hui et al. [36] have proposed some solutions based on HOSM. These studies highlight the capability of high-order sliding mode to effectively suppress chattering while maintaining robustness and anti-disturbance performance. In view of the advantages of HOSM, many scholars have tried to apply it in the control of ETVs in recent years. Reichhartinger et al. [37] applied the super-twisting technique to an electronic throttle valve controller and a state observer. Long et al. [38] proposed a controller for the electronic throttle (ET) system that incorporates a hierarchical two-layer sliding surface. Experimental results have demonstrated that the HOSM controllers significantly improve the effectiveness of electronic throttle valve control.

Based on the above analysis, this paper proposes an adaptive second-order fixed-time sliding mode (ASOFxTSM) controller for ETV. The controller incorporates a control law determined by two hierarchical sliding surfaces, offering both a fixed-time convergence guarantee and effective chattering suppression. Additionally, an adaptive mechanism based on a disturbance observer is introduced. Once the system converges to the vicinity of the origin, the coefficient of the switching term is progressively reduced, utilizing the minimum gain that ensures system stability. The performance of the proposed controller is evaluated through simulations and experiments, comparing it with other typical algorithms under two different conditions.

This paper makes several key contributions: 

(1) A novel adaptive second-order fixed-time convergent sliding mode controller is proposed, offering a fixed-time convergence guarantee and effective chattering suppression;

(2) An adaptive mechanism is devised, leveraging a fast-converging disturbance observer. This mechanism enables dynamic adjustment of control parameters, ensuring precise and efficient control under varying conditions;

(3) A stability analysis of the proposed controller is conducted, and the stable neighborhood of the system is determined.

The paper is structured as follows. Section 2 addresses the modeling of the electronic throttle valve, which accounts for parameter uncertainties. Section 3 introduces the FxTSM controller and the design method of high-order sliding mode. Then, the SOFxTSM controller and ASOFxTSM controller are designed. Section 4 presents comparative results obtained through simulations and experiments, along with corresponding numerical assessments. The conclusions of the research and discussion on the limitations are provided in Section 5.

## 2. Modeling of Electric Throttle Valve

**Figure 1:**
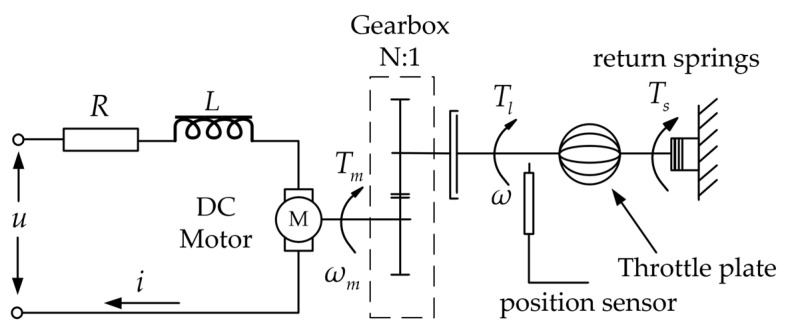
The structure of the ETV system.

The main components of the ETV system include a DC motor, a gearbox, a throttle plate, return springs, and a position sensor. Figure 1 illustrates the structural arrangement of the ETV system.

The mechanical equation of the throttle plate has the following form:

(1) $$J_{et}\dot{\omega }=T_{l}-T_{f}-T_{s}-T_{L}$$

where, *J_et_* is the rotational inertia of the throttle plate, ω is the angular velocity of the throttle plate, *T_l_*, *T_f_*, *T_s_*, and *T_L_* are the output torque of the gearbox, friction torque, reset spring torque, and an intake load torque of throttle plate, respectively. *T_L_* is typically influenced by the intake airflow and is considered a disturbance [39]. The following are the expressions of *T_f_* and *T_s_*:

(2) $$\left\{\begin{array}{l} T_{f}=k_{d}\omega +k_{k}\mathrm{sgn}(\omega )\\ T_{s}=k_{s}(\theta -\theta_{0})+k_{m}\mathrm{sgn}(\theta -\theta_{0}) \end{array}\right.$$

where, *θ* is the angle of the throttle plate, *θ*_0_ is the initial angle of the throttle plate, and *k_d_*, *k_k_*, *k_s_,* and *k_m_* are the viscous damping coefficient, coulomb friction coefficient, spring offset coefficient, and spring gain coefficient, respectively.

The electromechanical part of the DC motor is modeled as follows:

(3) $$\left\{\begin{array}{l} L\dot{i}=u-Ri-k_{e}\omega_{m}\\ J_{m}{\dot{\omega }}_{m}=k_{t}i-B_{m}\omega_{m}-T_{m} \end{array}\right.$$

where, *N* = *ω_m_/ω*, *ω_m_* is the angular velocity, *i* is the armature current, *u* is the control voltage, *k_e_* is the coefficient of electromotive force, *R* is the armature resistance, *J_m_* is the inertial moment, *k_t_* is the motor torque coefficient, *B_m_* is viscous damping coefficient, *T_m_* is the output torque. Due to the presence of gear backlash in the gearbox, the torque relationship between the motor and the throttle plate can be expressed as follows:

(4) $$T_{l}=NT_{m}+d(T_{m})$$

where, *d*(*T*_m_) is a bounded function of *T_m_*, satisfying |*T_m_*| ≤ *d_m_*, *d_m_ >* 0.

The value of inductance *L* being very small, allows for the neglect of dynamic change in current. Therefore, the ETV system model is obtained by combining (1)–(4):

(5) $$\delta_{10}\ddot{\theta }+\delta_{20}\dot{\theta }+\delta_{30}(\theta -\theta_{0})+\delta_{40}\mathrm{sgn}(\theta -\theta_{0})+\delta_{50}\mathrm{sgn}(\dot{\theta })-d=u$$

where,

$$d=\frac{R\left(d\left(T_{m}\right)-T_{L}\right)}{Nk_{t}},\;\delta_{10}=\frac{R\left(J_{et}+N^{2}J_{m}\right)}{Nk_{t}},\delta_{20}=\frac{\left(N^{2}k_{t}k_{e}+RN^{2}B_{m}+Rk_{d}\right)}{Nk_{t}},\delta_{30}=\frac{Rk_{s}}{Nk_{t}},\delta_{40}=\frac{Rk_{m}}{Nk_{t}},\delta_{50}=\frac{Rk_{k}}{Nk_{t}}$$

Considering the parameter uncertainty caused by the machining error and aging of the parts, (5) becomes

(6) $$(\delta_{10}+\Delta \delta_{1})\ddot{\theta }+(\delta_{20}+\Delta \delta_{2})\dot{\theta }+(\delta_{30}+\Delta \delta_{3})(\theta -\theta_{0})+(\delta_{40}+\Delta \delta_{4})\mathrm{sgn}(\theta -\theta_{0})+(\delta_{50}+\Delta \delta_{5})\mathrm{sgn}(\dot{\theta })-d=u$$

where, Δ*δ*_1_, Δ*δ*_2_, Δ*δ*_3_, Δ*δ*_4_, Δ*δ*_5_ are the uncertainties of the corresponding parameters. Those uncertainties and disturbances are written as lumped disturbances:

(7) $$\delta_{10}\ddot{\theta }+\delta_{20}\dot{\theta }+\delta_{30}{\left(\theta -\theta \right)}_{0}+\delta_{40}\mathrm{sgn}(\theta -\theta_{0})+\delta_{50}\mathrm{sgn}(\dot{\theta })=u+\Delta d$$

where, Δ*d* = − Δ*δ*_1_$\;\ddot{\theta }$ − Δ*δ*_2_$\;\dot{\theta }$ − Δ*δ*_3_(*θ* − *θ*_0_) − Δ*δ*_4_sgn(*θ* − *θ*_0_) − Δ*δ*_5_sgn($\dot{\theta }$) + *d*.

Define *φ* as the reference signal. Then, the system state variables *x*_1_ and *x*_2_ are defined as the errors of throttle opening value and angular velocity, respectively:

(8) $$x_{1}=\theta -\varphi ,\;x_{2}=\omega -\dot{\varphi }$$

Combining (7) and (8), the state equation of the ETV can be obtained as follows:

(9) $$\left\{\begin{array}{l} {\dot{x}}_{1}=x_{2}\\ {\dot{x}}_{2}=\mu u-\ddot{\varphi }-\mu \delta_{20}\dot{\theta }-\mu \delta_{30}(\theta -\theta_{0})-\mu \delta_{40}\mathrm{sgn}(\theta -\theta_{0})-\mu \delta_{50}\mathrm{sgn}(\dot{\theta })+d_{l}\\ y=x_{1} \end{array}\right.$$

where, *μ =* 1/*δ*_10_, *d_l_ = μ*Δ*d*.

## 3. Controller Design and Stability Analysis

### 3.1. Fixed-Time Sliding Mode Controller

To simplify the expression, we define the involution operation with the number’s sign as $x^{\left[a\right]}={\left|x\right|}^{a}\mathrm{sgn}\left(x\right),x\in R,a\in R$.

**Lemma 1**  [23]**.** *Consider a dynamic system:*

(10) $$\dot{y}=-\alpha_{1}y^{\left[\gamma_{1}\right]}-\alpha_{2}y-\alpha_{3}y^{\left[\gamma_{2}\right]},y(0)=y_{0}$$

*where, α_1_ > 0, α_2_ > 0, α_3_ > 0, γ_1_ > 1, 0 < γ_2_ < 1. Then, this dynamic establishes fixed-time stability at the equilibrium point of the system and bounds the settling time as follows*:

(11) $$T_{\max}=\frac{1}{\alpha_{2}}\left[\frac{1}{\left(\gamma_{1}-1\right)}\ln\left(1+\frac{\alpha_{2}}{\alpha_{1}}\right)+\frac{1}{\left(1-\gamma_{2}\right)}\ln\left(1+\frac{\alpha_{2}}{\alpha_{3}}\right)\right]$$

The rigorous proof of Lemma 1 can be found in reference [23]. For the second-order nonlinear system (9), the terminal sliding surface with a fixed-time convergence guarantee is designed by combining Lemma 1:

(12) $$s=x_{2}+\alpha_{1}x_{1}{}^{\left[\gamma_{1}\right]}+\alpha_{2}x_{1}+\alpha_{3}x_{1}{}^{\left[\gamma_{2}\right]}$$

where, *α*_1_ > 0, *α*_2_ > 0, *α*_3_ > 0, *γ*_1_ > 1, 0 < *γ*_2_ < 1. A fixed-time convergence reaching law is adopted for *s*:

(13) $$\dot{s}=-a_{1}s^{\left[b_{1}\right]}-a_{2}s-a_{3}s^{\left[b_{2}\right]}-K_{1}\mathrm{sgn}(s)$$

where, *a*_1_ > 0, *a*_2_ > 0, *a*_3_ > 0, *K*_1_ > |*d*_l_|, *b*_1_ > 1, 0 < *b*_2_ < 1. The system state Equation (9), sliding surface (12), and dynamic (13) are combined to obtain the control output of the fixed-time sliding mode (FxTSM) controller:

(14) $$u=-\mu^{-1}\left[\left(\alpha_{1}\gamma_{1}{\left|x_{1}\right|}^{\gamma_{1}-1}+\alpha_{2}+\alpha_{3}\gamma_{2}{\left|x_{1}\right|}^{\gamma_{2}-1}\right)x_{2}+f(x)+a_{1}s^{\left[b_{1}\right]}+a_{2}s+a_{3}s^{\left[b_{2}\right]}+K_{1}\mathrm{sgn}(s)\right]$$

where, *f*(*x*) = −$\ddot{\varphi }$− *μδ*_20_$\;\dot{\theta }$ − *μδ*_30_(*θ*−*θ*_0_) − *μδ*_40_sgn(*θ* − *θ*_0_) − *μδ*_50_sgn($\dot{\theta }$).

**Remark 1.**  *The convergence of the FxTSM controller is proved in [23] and it is concluded that the settling time solely depends on the controller parameters. Since the function y(x) = ln (1 + x)/x is a monotonically decreasing function, combined with the settling time expression in Lemma 1, it can be seen that as α_2_ and a_2_ increase, the system settling time will reduce.*

**Remark 2.**  *In the pursuit of faster convergence, FxTSM incorporates the power terms. However, in practical applications, when the system experiences significant fluctuations near the origin, the large power term directly affects the control output u, leading to considerable chattering that cannot be fully addressed by FxTSM alone. Research in [40] highlights that enhancing control continuity is a crucial approach to reducing chattering, which is a distinctive advantage of high-order sliding mode*.

### 3.2. Second-Order Fixed-Time Sliding Mode Controller

For system 9, the objective of the traditional sliding mode approach is to maintain *s* at zero while the control *u* appears in $\dot{s}$, as explained in Section 3.1. However, the chattering effect often makes the solution unacceptable. To counteract chattering, one approach is to treat the control derivative $\dot{u}$ as a virtual control variable [41]. Usually, the sliding function *σ* = *s* + $\dot{s}$ is chosen. Consequently, $\dot{\sigma }=\dot{s}+\ddot{s}$ will encompass the new control output, and *σ* can be regulated to zero using classic sliding mode strategies. Then, *s* gradually tends towards zero. This constitutes the foundational principle of the second-order sliding mode design.

Based on the above analysis, a new variable *σ* is introduced to ensure that the system state can converge to the sliding surface *s* = 0 in fixed time. Then, the *σ* sliding surface with fixed-time stability can be designed as

(15) $$\sigma =\dot{s}+\beta_{1}s^{\left[\varepsilon_{1}\right]}+\beta_{2}s+\beta_{3}s^{\left[\varepsilon_{2}\right]}$$

where, *β*_1_ > 0, *β*_2_ > 0, *β*_3_ > 0, *ε*_1_ > 1, 0 < *ε*_2_ < 1. Correspondingly, a reaching law with fixed-time stability is designed for *σ*:

(16) $$\dot{\sigma }=-k_{1}\sigma^{\left[\eta_{1}\right]}-k_{2}\sigma -k_{3}\sigma^{\left[\eta_{2}\right]}-K_{2}\mathrm{sgn}(\sigma )$$

where, *k*_1_ > 0, *k*_2_ > 0, *k*_3_ > 0, *η*_1_ > 1, 0 < *η*_2_ < 1, *K*_2_ > 0. 

Due to the introduction of the *σ* sliding surface, the switching term does not directly act on control *u* but the virtual control $\dot{u}$. Hence, the effect of the switching term becomes continuous through the influence of the integral term, ultimately resolving the chattering issue. Then it is the solution to the virtual control $\dot{u}$, and the expression of the control output *u* is further obtained.

By deriving the sliding mode surface *s* (12), the formula for controlling *u* is obtained as:

(17) $$\dot{s}=f(x)+\mu u+d_{l}+\left(\alpha_{1}\gamma_{1}{\left|x_{1}\right|}^{\gamma_{1}-1}+\alpha_{2}+\alpha_{3}\gamma_{2}{\left|x_{1}\right|}^{\gamma_{2}-1}\right)x_{2}$$

Based on the equivalent control design method [42], control *u* is divided into two parts, including the equivalent control and the switching control:

(18) $$u=u_{eq}+u_{s}$$

The equivalent control is a feedforward term related to the system state, which can compensate for the influence of the nonlinear factors of the system:

(19) $$u_{eq}=-\mu^{-1}f(x)$$

Substituting the compensated control *u* (18) into (17),

(20) $$\dot{s}=\mu u_{s}+d_{l}+\left(\alpha_{1}\gamma_{1}{\left|x_{1}\right|}^{\gamma_{1}-1}+\alpha_{2}+\alpha_{3}\gamma_{2}{\left|x_{1}\right|}^{\gamma_{2}-1}\right)x_{2}$$

By deriving (20), the formula for the virtual control $\dot{u}$ is obtained as

(21) $$\begin{array}{ll} \ddot{s} & =\mu {\dot{u}}_{s}+{\dot{d}}_{l}+\left(\alpha_{1}\gamma_{1}{\left|x_{1}\right|}^{\gamma_{1}-1}+\alpha_{2}+\alpha_{3}\gamma_{2}{\left|x_{1}\right|}^{\gamma_{2}-1}\right){\dot{x}}_{2}+\\ & \left(\alpha_{1}\gamma_{1}\left(\gamma_{1}-1\right)x_{1}{}^{\left[\gamma_{1}-2\right]}+\alpha_{3}\gamma_{2}\left(\gamma_{2}-1\right)x_{1}{}^{\left[\gamma_{2}-2\right]}\right)x_{2}^{2}\\ & =\mu {\dot{u}}_{s}+{\dot{d}}_{l}+\left(\alpha_{1}\gamma_{1}{\left|x_{1}\right|}^{\gamma_{1}-1}+\alpha_{2}+\alpha_{3}\gamma_{2}{\left|x_{1}\right|}^{\gamma_{2}-1}\right)(\mu u_{s}+d_{l})+\\ & \left(\alpha_{1}\gamma_{1}\left(\gamma_{1}-1\right)x_{1}{}^{\left[\gamma_{1}-2\right]}+\alpha_{3}\gamma_{2}\left(\gamma_{2}-1\right)x_{1}{}^{\left[\gamma_{2}-2\right]}\right)x_{2}^{2} \end{array}$$

After taking the derivative of the sliding surface *σ* and substituting the expressions of $\dot{s}$ (20) and $\ddot{s}$ (21) into it, the following expression is obtained:

(22) $$\begin{array}{ll} \dot{\sigma } & =\ddot{s}+\left(\beta_{1}\varepsilon_{1}{\left|s\right|}^{\varepsilon_{1}-1}+\beta_{2}+\beta_{3}\varepsilon_{2}{\left|s\right|}^{\varepsilon_{2}-1}\right)\dot{s}\\ & =\mu {\dot{u}}_{s}+{\dot{d}}_{l}+w_{1}(\mu u_{s}+d_{l})+\left[\alpha_{1}\gamma_{1}\left(\gamma_{1}-1\right)x_{1}{}^{\left[\gamma_{1}-2\right]}+\alpha_{3}\gamma_{2}\left(\gamma_{2}-1\right)x_{1}{}^{\left[\gamma_{2}-2\right]}\right]x_{2}^{2}\\ & +w_{2}(\mu u_{s}+d_{l})+w_{1}w_{2}x_{2}\\ & =\mu {\dot{u}}_{s}+{\dot{d}}_{l}+(w_{1}+w_{2})(\mu u_{s}+d_{l})+g(x) \end{array}$$

where, *w*_1_ = *α*_1_*γ*_1_${\left|x_{1}\right|}^{\gamma_{1}-1}$ + *α*_2_ + *α*_3_*γ*_2_${\left|x_{1}\right|}^{\gamma_{2}-1}$, *w*_2_ = *β*_1_*ε*_1_${\left|s\right|}^{\varepsilon_{1}-1}$ + *β*_2_ + *β*_3_*ε*_2_${\left|s\right|}^{\varepsilon_{2}-1}$, *g*(*x*) = *w*_1_*w*_2×2_ + *α*_1_*γ*_1_(*γ*_1_ − 1)$x_{1}{}^{\left[\gamma_{1}-2\right]}x_{2}^{2}$ + *α*_3_*γ*_2_(*γ*_2_ − 1)$x_{1}{}^{\left[\gamma_{2}-2\right]}x_{2}^{2}$.

In order to facilitate the solution of the virtual control ${\dot{u}}_{s}$, the equivalent control design method is also adopted. The control ${\dot{u}}_{s}$ is divided into two parts, including the equivalent control and the switching control:

(23) $${\dot{u}}_{s}={\dot{u}}_{seq}+{\dot{u}}_{switch}$$

The equivalent control here is related to both the system state and the sliding surface:

(24) $${\dot{u}}_{seq}=-\mu^{-1}\left[\left(w_{1}+w_{2})\mu u_{seq}+g(x\right)\right]$$

Combining with reaching law (16), the switching control ${\dot{u}}_{switch}$ is designed as

(25) $${\dot{u}}_{switch}=-\mu^{-1}\left(k_{1}\sigma^{\left[\eta_{1}\right]}+k_{2}\sigma +k_{3}\sigma^{\left[\eta_{2}\right]}+K_{2}\mathrm{sgn}(\sigma )\right)$$

The final expression of the control *u* can be obtained by combining (18) and (23–25):

(26) $$\begin{array}{ll} u & =u_{eq}+\int {\dot{u}}_{seq}+\int {\dot{u}}_{switch}\\ & =u_{eq}+\int {\dot{u}}_{seq}+\mu^{-1}\int \left(-k_{1}\sigma^{\left[\eta_{1}\right]}-k_{2}\sigma -k_{3}\sigma^{\left[\eta_{2}\right]}-K_{2}\mathrm{sgn}(\sigma )\right) \end{array}$$

**Theorem 1.**  *Consider the second-order nonlinear system (9), if the control output is given by (26) and the control parameter K_2_ satisfies K_2_ > |(w_1_ + w_2_)d_l_ +* ${\dot{d}}_{l}$*|. Subsequently, the system state will approach a neighborhood of the origin in fixed time, with the settling time T(x_0_) bounded by*:

(27) $$T(x_{0})\leq T_{\max}$$

*where, T_max_ = T_σmax_ + T_smax_ + T_rmax_,*

$$T_{\sigma \max}=\frac{1}{k_{2}}\left[\frac{1}{\left(\eta_{1}-1\right)}\ln\left(1+\frac{k_{2}}{k_{1}}\right)+\frac{1}{\left(1-\eta_{2}\right)}\ln\left(1+\frac{k_{2}}{k_{3}}\right)\right],$$

$$T_{s\max}=\frac{1}{\beta_{2}}\left[\frac{1}{\left(\varepsilon_{1}-1\right)}\ln\left(1+\frac{\beta_{2}}{\beta_{1}}\right)+\frac{1}{\left(1-\varepsilon_{2}\right)}\ln\left(1+\frac{\beta_{2}}{\beta_{3}}\right)\right],$$

$$T_{r\max}=\frac{1}{\alpha_{2}}\left[\frac{1}{\left(\gamma_{1}-1\right)}\ln\left(1+\frac{\alpha_{2}}{\alpha_{1}}\right)+\frac{1}{\left(1-\gamma_{2}\right)}\ln\left(1+\frac{\alpha_{2}}{\alpha_{3}}\right)\right].$$

**Proof of Theorem 1.**  The Lyapunov function is constructed as

(28) $$V_{1}=\frac{1}{2}\sigma^{2}$$

The time derivative of *V*_1_ is

(29) $$\begin{array}{ll} {\dot{V}}_{1} & =\sigma \dot{\sigma }\\ & =\sigma \left[\ddot{s}+\left(\beta_{1}\varepsilon_{1}s^{\varepsilon_{1}-1}+\beta_{2}+\beta_{3}\varepsilon_{2}s^{\varepsilon_{2}-1}\right)\dot{s}\right]\\ & =\sigma \left[\left(w_{1}+w_{2})(\mu u_{S}+d_{l})+\mu {\dot{u}}_{S}+{\dot{d}}_{l}+g(x\right)\right]\\ & =\left|\sigma \right|\left(-k_{1}{\left|\sigma \right|}^{\eta_{1}}-k_{2}\left|\sigma \right|-k_{3}{\left|\sigma \right|}^{\eta_{2}}\right)-\left|\sigma \right|\left|K_{2}-(w_{1}+w_{2})d_{l}-{\dot{d}}_{l}\right|\\ & \leq \left|\sigma \right|\left(-k_{1}{\left|\sigma \right|}^{\eta_{1}}-k_{2}\left|\sigma \right|-k_{3}{\left|\sigma \right|}^{\eta_{2}}\right)\\ & =-2k_{1}V_{1}^{\frac{\eta_{1}+1}{2}}-2k_{2}V_{1}-2k_{3}V_{1}^{\frac{\eta_{2}+1}{2}} \end{array}$$

Combining with Lemma 1, it can be concluded that this dynamic guarantees fixed-time stability and bounds the settling time as follows:

(30) $$T_{\sigma \max}=\frac{1}{k_{2}}\left[\frac{1}{\left(\eta_{1}-1\right)}\ln\left(1+\frac{k_{2}}{k_{1}}\right)+\frac{1}{\left(1-\eta_{2}\right)}\ln\left(1+\frac{k_{2}}{k_{3}}\right)\right]$$

Then, the system state will reach the sliding surface *σ* = 0, and the ideal sliding trajectory of the system can be obtained by combining with (15):

(31) $$\dot{s}=-\beta_{1}s^{\left[\varepsilon_{1}\right]}-\beta_{2}s-\beta_{3}s^{\left[\varepsilon_{2}\right]}$$

When the system trajectory adheres to (31), it will reach the sliding surface *s* = 0 within fixed time, and the settling time is bounded by:

(32) $$T_{s\max}=\frac{1}{\beta_{2}}\left[\frac{1}{\left(\varepsilon_{1}-1\right)}\ln\left(1+\frac{\beta_{2}}{\beta_{1}}\right)+\frac{1}{\left(1-\varepsilon_{2}\right)}\ln\left(1+\frac{\beta_{2}}{\beta_{3}}\right)\right]$$

When the system state reaches the sliding surface *s* = 0, the ideal sliding trajectory of the system can be obtained by combining (12):

(33) $$x_{2}={\dot{x}}_{1}=-\alpha_{1}x_{1}{}^{\left[\gamma_{1}\right]}-\alpha_{2}x_{1}-\alpha_{3}x_{1}{}^{\left[\gamma_{2}\right]}$$

Combining with Lemma 1, it can be concluded that this dynamic guarantees fixed-time stability and bounds the settling time as follows:

(34) $$T_{r\max}=\frac{1}{\alpha_{2}}\left[\frac{1}{\left(\gamma_{1}-1\right)}\ln\left(1+\frac{\alpha_{2}}{\alpha_{1}}\right)+\frac{1}{\left(1-\gamma_{2}\right)}\ln\left(1+\frac{\alpha_{2}}{\alpha_{3}}\right)\right]$$

The proof of Theorem 1 is now concluded. □

**Remark 3.**  *The proof process demonstrates that the value of K_2_ depends on* $\dot{d}$*, and the challenge of determining the upper bound*$\dot{d}$*leads to the overestimation of K_2_. To address this, an adaptive method is introduced to determine the most suitable value of K_2_.*

### 3.3. Adaptive Second-Order Fixed-Time Sliding Mode Controller

For the second-order system (9), the fixed-time disturbance-observer (FxTDO) is formulated as

(35) $$\left\{\begin{array}{l} {\dot{z}}_{1}=l_{1}h_{1}\left(k_{o}{\tilde{x}}_{2}\right)+z_{2}+f(x)+\mu u\\ {\dot{z}}_{2}=l_{2}h_{2}\left(k_{o}{\tilde{x}}_{2}\right) \end{array}\right.$$

where, *z*_1_ and *z*_2_ are state variables of FxTDO and they are utilized to estimate the system state *x*_2_ and lumped disturbance *d_l_*, respectively, ${\tilde{x}}_{2}$ = $x_{2}$ − *z*_1_ is differentiation error, *k_o_* is the error amplification coefficient and satisfies *k_o_* > 0, $l_{1}>0$, $l_{2}>0$, $l_{1}\leq 2\sqrt{l_{2}}$, $h_{1}\left(x\right)=\left(x^{\left[\kappa_{1}\right]}+x^{\left[\kappa_{2}\right]}\right)$, $h_{2}\left(x\right)=\left(x^{\left[2\kappa_{1}-1\right]}+x^{\left[2\kappa_{2}-1\right]}\right)$, $\kappa_{1}\in \left(0.5,1\right)$, $\kappa_{2}\in \left(1,1.5\right)$.

**Lemma 2**  [43]**.** *Consider the second-order nonlinear system (9) with a fixed-time disturbance observer (35), assuming that the lumped disturbance satisfies the boundary condition |d_l_| ≤ τ, where τ > 0, the observation error |d_l_ – z_2_| will converge to a neighborhood of the origin in fixed time, that is,* ∃E>0*, |d_l_ – z_2_| ≤ E,* $\forall t\geq T_{d\max}$*, the settling time T_dmax_ has the following expression*:

(36) $$T_{d\max}=\frac{1}{c}\left(\frac{2\kappa_{1}}{1-\kappa_{1}}+\frac{2\kappa_{2}}{\kappa_{2}-1}\right)$$

*where, c is a positive number, and E is the tracking error of FxTDO.*

The expression of *E* is given in [43], and *E* decreases with the increase of *k_o_*. Therefore, the control *u* is redesigned as

(37) $$\left\{\begin{array}{l} u=u_{eq0}+u_{s}\\ u_{eq0}=-\mu^{-1}\left(f(x)+z_{2}\right) \end{array}\right.$$

Substituting (37) into (17), we obtain

(38) $$\dot{s}=\mu u_{s0}+d_{l}-z_{2}+w_{1}x_{2}$$

In equation (38), the lumped disturbance *d_l_* is compensated by *z*_2_, and it is represented as the observation error *E*. Therefore, $\ddot{s}$ will not include the term ${\dot{d}}_{l}$ and can be expressed as follows:

(39) $$\begin{array}{ll} \ddot{s} & =\mu {\dot{u}}_{s}+{\dot{d}}_{l}-{\dot{z}}_{2}+w_{1}{\dot{x}}_{2}+\left[\alpha_{1}\gamma_{1}\left(\gamma_{1}-1\right)x_{1}{}^{\left[\gamma_{1}-2\right]}+\alpha_{3}\gamma_{2}\left(\gamma_{2}-1\right)x_{1}{}^{\left[\gamma_{2}-2\right]}\right]x_{2}^{2}\\ & =\mu {\dot{u}}_{s}+{\dot{d}}_{l}-{\dot{z}}_{2}+w_{1}\left(\mu u_{s}+d_{l}-z_{2}\right)+\left[\alpha_{1}\gamma_{1}\left(\gamma_{1}-1\right)x_{1}{}^{\left[\gamma_{1}-2\right]}+\alpha_{3}\gamma_{2}\left(\gamma_{2}-1\right)x_{1}{}^{\left[\gamma_{2}-2\right]}\right]x_{2}^{2} \end{array}$$

Take the derivative of the sliding surface *σ* and substitute the expressions of $\dot{s}$ (38) and $\ddot{s}$ (39) into it:

(40) $$\begin{array}{ll} \dot{\sigma } & =\ddot{s}+w_{2}\dot{s}\\ & =\mu {\dot{u}}_{s}+{\dot{d}}_{l}-{\dot{z}}_{2}+(w_{1}+w_{2})(\mu u_{s}+d_{l}-z_{2})+g(x) \end{array}$$

Similarly, the virtual control ${\dot{u}}_{s}$ is divided into two parts, including the equivalent control and the switching control:

(41) $${\dot{u}}_{s}={\dot{u}}_{seq0}+{\dot{u}}_{switch0}$$

The equivalent control here is similar to (24):

(42) $${\dot{u}}_{seq0}=-\mu^{-1}\left[\left(w_{1}+w_{2})\mu u_{seq0}+g(x\right)\right]$$

Combining with reaching law (16), the switching control is designed as

(43) $${\dot{u}}_{switch0}=-\mu^{-1}\left(k_{1}\sigma^{\left[\eta_{1}\right]}+k_{2}\sigma +k_{3}\sigma^{\left[\eta_{2}\right]}+K_{3}\mathrm{sgn}(\sigma )\right)$$

The final expression of the control *u* can be obtained by combining (37) and (40)–(42):

(44) $$\begin{array}{ll} u & =u_{eq0}+\int {\dot{u}}_{seq0}+\int {\dot{u}}_{switch0}\\ & =u_{eq0}+\int {\dot{u}}_{seq0}+\mu^{-1}\int \left(-k_{1}\sigma^{\left[\eta_{1}\right]}-k_{2}\sigma -k_{3}\sigma^{\left[\eta_{2}\right]}-K_{3}\mathrm{sgn}(\sigma )\right) \end{array}$$

Since *E* is a neighborhood of the origin, it can be assumed that $\left|{\dot{d}}_{l}-{\dot{z}}_{2}\right|\leq M,M>0$ without loss of generality. In order to adopt the minimum value of *K*_3_ after achieving convergence, an adaptive mechanism is designed to adjust the value of *K*_3_. Define a neighborhood ***E****_f_* of *σ* = 0, where the neighborhood is $E_{f}=\{\sigma \in R|V(\sigma )\leq V_{2-f},V_{2-f}>0\}$. When the system state is far away from ***E****_f_*, *K*_3_ takes the maximum value to ensure that the system state can converge to ***E****_f_* with the expected performance. When the system state converges into ***E****_f_*, an adaptive mechanism is designed:

(45) $$K_{3}=\left\{\begin{array}{l} K_{a}\;,\;V(\sigma )\geq V_{2-f}\\ K_{i}\;,\;V(\sigma )<V_{2-f} \end{array}\right.$$

where, *V*(*σ*) = 1/2*σ*^2^, *K_a_* ≥ *M* + max|(*w*_1_ + *w*_2_)*E*|, ${\dot{K}}_{i}=-qV$, *K_i_*(0) = *K_a_*, *q* > 0.

**Theorem 2.**  *Consider the second-order nonlinear system (9), if the control output is given by (44). Then the system state will converge to a neighborhood of the origin in fixed time and maintain a stable state. The settling time T_1_(x_0_) is bounded by*

(46) $$T_{1}(x_{0})\leq T_{\max}$$

*where, T_max_ = T_σmax_ + T_smax_ + T_rmax_, the specific expression of the convergence time is the same as Theorem 1.*

**Proof of Theorem 2.**  The Lyapunov function is constructed as

(47) $$V_{2}=\frac{1}{2}\sigma^{2}$$

The time derivative of *V*_2_ is

(48) $$\begin{array}{ll} {\dot{V}}_{2} & =\sigma \dot{\sigma }\\ & =\sigma \left[\ddot{s}+\left(\beta_{1}\varepsilon_{1}s^{\varepsilon_{1}-1}+\beta_{2}+\beta_{3}\varepsilon_{2}s^{\varepsilon_{2}-1}\right)\dot{s}\right]\\ & =\sigma \left(\mu {\dot{u}}_{s}+{\dot{d}}_{l}-{\dot{z}}_{2}+(w_{1}+w_{2})(\mu u_{s}+d_{l}-z_{2})+g(x)\right)\\ & =\sigma \left(-k_{1}\sigma^{\left[\eta_{1}\right]}-k_{2}\sigma -k_{3}\sigma^{\left[\eta_{2}\right]}-K_{2}\mathrm{sgn}(\sigma )+{\dot{d}}_{l}-{\dot{z}}_{2}+(w_{1}+w_{2})(d_{l}-z_{2})\right) \end{array}$$

According to Lemma 2, when *t* > *t_dmax_*, there exist |*d_l_* − *z*_2_| ≤ *E*. Then, (48) becomes

(49) $$\begin{array}{ll} {\dot{V}}_{2} & \leq \sigma \left[-k_{1}\sigma^{\left[\eta_{1}\right]}-k_{2}\sigma -k_{3}\sigma^{\left[\eta_{2}\right]}-K_{3}\mathrm{sgn}(\sigma )+{\dot{d}}_{l}-{\dot{z}}_{2}+(w_{1}+w_{2})(d_{l}-z_{2})\right]\\ & \leq \sigma \left(-k_{1}\sigma^{\left[\eta_{1}\right]}-k_{2}\sigma -k_{3}\sigma^{\left[\eta_{2}\right]}\right)-\sigma \left[K_{3}\mathrm{sgn}(\sigma )-M-(w_{1}+w_{2})E\right] \end{array}$$

When the system state does not enter ***E****_f_*, *K*_3_ adopts its maximum value, i.e., *K*_3_ = *K_a_*, and then (49) becomes

(50) $${\dot{V}}_{2}\leq -2k_{1}V_{2}^{\frac{\eta_{1}+1}{2}}-2k_{2}V_{2}-2k_{3}V_{2}^{\frac{\eta_{2}+1}{2}}$$

According to Lemma 1, the system state will reach the sliding surface *σ* = 0 in fixed time, and the settling time has the following upper bound:

(51) $$T_{\sigma \max}=\frac{1}{k_{2}}\left[\frac{1}{\left(\eta_{1}-1\right)}\ln\left(1+\frac{k_{2}}{k_{1}}\right)+\frac{1}{\left(1-\eta_{2}\right)}\ln\left(1+\frac{k_{2}}{k_{3}}\right)\right]$$

Once the system state enters ***E****_f_*, *K*_3_ transitions into an adaptive parameter, i.e., *K*_3_ = *K_i_*, then (49) becomes

(52) $${\dot{V}}_{2}=\sigma \left[-k_{1}\sigma^{\left[\eta_{1}\right]}-k_{2}\sigma -k_{3}\sigma^{\left[\eta_{2}\right]}-K_{i}\mathrm{sgn}(\sigma )+{\dot{d}}_{l}-{\dot{z}}_{2}+(w_{1}+w_{2})(d_{l}-z_{2})\right]$$

Since the expected convergence region of the system is as small as possible, it can be assumed *V*_2−*f*_ < 1, then (52) becomes

(53) $${\dot{V}}_{2}\leq -2k_{3}V_{2}^{\frac{\eta_{2}+1}{2}}-K_{i}V_{2}^{\frac{1}{2}}+\left[M+(w_{1}+w_{2})E\right]V_{2}^{\frac{1}{2}}$$

Introduce an auxiliary function: $B\left(V_{2}\right)=2k_{3}V_{2}^{\frac{\eta_{2}}{2}}+K_{i}$, if $B\left(V_{2}\right)\geq M+(w_{1}+w_{2})E$, i.e., $V_{2}\geq \frac{1}{2k_{3}}{\left[-K_{i}+M+(w_{1}+w_{2})E\right]}^{\frac{-\eta_{2}}{2}}≜V_{2-b}$, (53) can reduce as

(54) $${\dot{V}}_{2}\leq -V_{2}^{\frac{1}{2}}\left[B\left(V_{2}\right)-M-(w_{1}+w_{2})E\right]\leq 0$$

Therefore, the system will reach the neighborhood ***E****_s_*, where $E_{s}=\{\sigma \in R|V(\sigma )\leq V_{2-b}\}$. Obviously, *V*_2−*b*_ ≤ *V*_2−*f*_, i.e., $E_{s}\in E_{f}$. So, the system will reach the neighborhood *V*_2−*f*_ in fixed time and be stable. When the system state reaches the neighborhood of *σ* = 0, the subsequent proof process is consistent with Proof 1, and the proof of Theorem 2 is thus completed. □

**Remark 4.**  *In order to ensure that it converges to the origin at a faster speed, K_3_ takes the maximum value before arriving at the neighborhood V_2−f_. The reason for designing K_3_ in sections is that when the system reaches a stable state, K_3_ is overvalued most of the time. So, the adaptive design of K_3_ is designed to use the minimum K_i_ to satisfy the stability of the system.*

**Figure 2:**
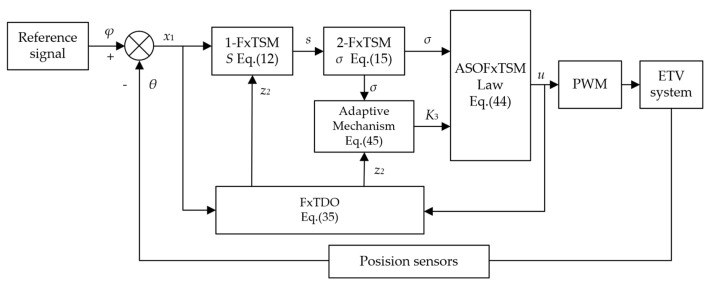
The block diagram of the ASOFxTSM controller.

The block diagram of the proposed controller is shown in Figure 2. Its core design includes two hierarchical fixed-time sliding surfaces and an adaptive mechanism based on the disturbance estimator.

## 4. Simulation and Experiment Validation

### 4.1. Simulation Setup

The control-oriented model of the electronic throttle valve is established based on BOSCH028750156 which was produced by Bosch. Its parameters are measured by the experimental method in [44] and are shown in Table 1.

External disturbances and parameter uncertainties are introduced into the simulation by using multiple harmonics:

(55) $$d_{l}\left(t\right)=\sin\left(15t\right)+0.7\sin\left[19\left(t+4\right)\right]+0.4\sin\left[28\left(t+17\right)\right]+0.08\sin\left(500t\right)+0.03\sin\left(800t\right)\left[Nm\right]$$

Three different working conditions are designed to verify the performance of the adaptive second-order fixed-time sliding mode(ASOFxTSM) controller proposed in this paper. To demonstrate the advantages of the proposed controller, a comparison is made with the fixed-time sliding mode (FxTSM) controller in [23] and the super-twisting controller in [45]. The super-twisting controller selects a linear sliding surface *s* = *x*_2_ + *c*_0_*x*_1_, and the control output is expressed as

(56) $$\left\{\begin{array}{l} u=-\lambda_{1}{\left|s\right|}^{1/2}\mathrm{sgn}(s)+v\\ \dot{v}=-\lambda_{2}\mathrm{sgn}(s) \end{array}\right.$$

where, *λ*_1_ > 0, *λ*_2_ > 0. 

Based on the nominal values of the ETV system parameters (as shown in Table 1) and the upper bound of lumped disturbance, the parameters of the ASOFxTSM controller were set and listed in Table 2. For FxTSM controller, the parameters were set as follows: *γ*_1_ = 7/5, *γ*_2_ = 5/9, *α*_1_ = 15, *α*_2_ = 15, *α*_3_ = 10, b_1_ = 7/5, b_1_ = 5/9, *a*_1_ = 20, *a*_2_ = 50, *a*_3_ = 30, *K*_1_ = 400. For the super-twisting controller, the parameters were set as follows: *c*_0_ = 50, *λ*_1_ = 220, *λ*_2_ = 2500.

To quantify the differences between the individual controllers, several criteria are utilized to compare the performance of the controlled system. Firstly, the maximum error (max*E*) is introduced, which represents the maximal deviation within the specified interval:

(57) $$\max E=\max\left|x_{1}\left(i\right)\right|$$

The Mean Absolute Error (MAE) needs to be introduced to reflect the average performance:

(58) $$\mathrm{MAE}=\frac{1}{n}\sum_{i=1}^{n}\left|x_{1}\left(i\right)\right|$$

Further, the Mean Square Error (MSE) was used:

(59) $$\mathrm{MSE}=\frac{1}{n}\sum_{i=1}^{n}x_{1}^{2}\left(i\right)$$

The above criteria can well reflect the performance of the controlled system. In order to evaluate the difference in controllers’ behavior, it is necessary to introduce the activity of control effort (CoEf) [5] for evaluation:

(60) $$\mathrm{CoEf}=\frac{1}{n}\sum_{i=1}^{n}\left|u_{i}-u_{0}\right|$$

where, *u*_0_ = *k_s_*(*θ* – *θ*_0_) + *k_m_*sgn(*θ* – *θ*_0_).

### 4.2. Simulation Verification

#### 4.2.1. Trajectory Tracking of Steps (Case 1)

**Figure 3:**
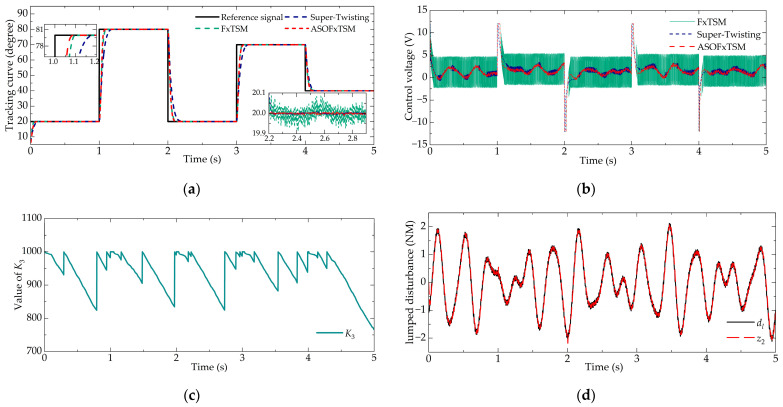
Tracking Performance in Case 1: (**a**) tracking curve, (**b**) control voltage, (**c**) value of *K*_3_, and (**d**) disturbance estimation.

In engine speed control strategies, the throttle valve angle is correlated with the torque demand. During engine startup or sudden load increase, a substantial torque is required, necessitating the rapid achievement of the predetermined throttle valve angle. A step signal is designed to assess the response capability and control accuracy of the throttle valve under these extreme conditions. The numerical simulation results are shown in Figure 3.

Figure 3a clearly illustrates that ASOFxTSM exhibits a convergence speed comparable to that of FxTSM. Take the case when response to a step signal from 20 to 80 deg, the settling time of ASOFxTSM is fastest with 88 ms followed by 95 ms for FxTSM and 175 ms for super-twisting. It is worth noting that since the super-twisting controller achieves asymptotic convergence, its settling time is affected by the initial state. For instance, the settling time is 117 ms when the reference signal changes from 70 to 40 deg and significantly extends to 175 ms when the reference signal changes from 20 to 80 deg. In contrast, ASOFxTSM has a fixed-time convergence property and maintains a consistently faster convergence speed under various conditions. Its convergence time is 86 ms when the reference signal changes from 70 to 40 deg and 88 ms when the reference signal changes from 20 to 80 deg. 

In theory, FxTSM may be considered to have the fastest convergence time. However, in practice, a more conservative parameter setting is required to account for the output limit and chattering reduction. As shown in Figure 3b, despite adopting smaller parameters, the output of FxTSM exhibited significant chattering, and this chattering of output resulted in a decrease in control accuracy. From Figure 3a, it is evident that ASOFxTSM achieved higher accuracy, followed by super-twisting, and FxTSM exhibited the lowest accuracy. Numerically, the MAE of ASOFxTSM, super-twisting, and FxTSM were 2.825 × 10^−3^, 5.438 × 10^−3^, and 28.497 × 10^−3^, respectively. These demonstrate the excellent chattering suppression capability of the proposed controller and the contribution to enhancing control accuracy. 

Figure 3c shows that the designed adaptive mechanism can effectively reduce the value of *K*_3_ once the system reaches a stable state. Additionally, Figure 3d demonstrates that the adopted observer exhibits improved tracking performance for disturbances. When encountering large changes in the target value, minor fluctuations are observed. This is attributed to the error amplification factor *k_o_* within the observer, which provides sufficient output to compensate for significant errors.

It is worth noting that the integral switching term of super-twisting and the absence of a fast convergence term result in its inability to promptly compensate for large disturbances. In other words, while a super-twisting controller can produce a smooth output, it comes at the cost of robustness, which can also impact steady-state accuracy. As a consequence, when facing substantial disturbances, the control accuracy of super-twisting decreases, as evident from the increase in the values of max and MSE. For the convenience of comparison, the steady-state criteria are listed in Table 3 and it can be seen that the ASO controller has the best steady-state performance.

Parameter CoEf in Table 3 represents the control output performance of the three controllers. FxTSM had significantly larger values (1.5406), while ASOFxTSM had the smallest values (0.5381) among the three controllers. This indicates that the proposed ASOFxTSM controller is more efficient in terms of control efforts and requires the least energy to meet the control requirements. These attributes make ASOFxTSM a highly promising and energy-efficient solution for the ETV system.

#### 4.2.2. Trajectory Tracking of Sinusoidal (Case 2)

**Figure 4:**
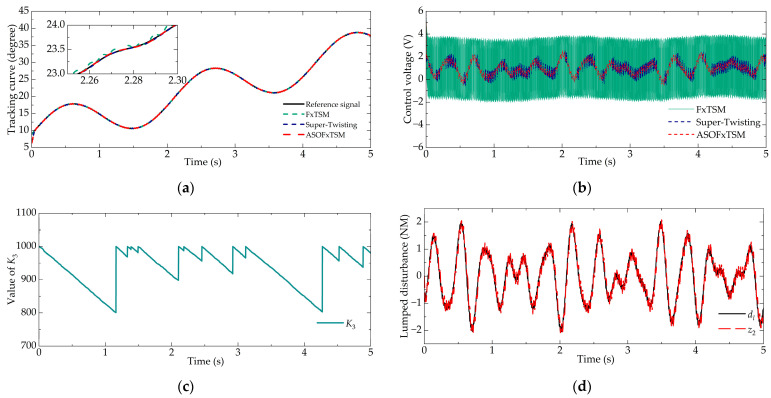
Tracking performance in Case 2: (**a**) tracking curve, (**b**) control voltage, (**c**) value of *K*_3_, and (**d**) disturbance estimation.

Achieving precise tracking control of the throttle valve is crucial. To evaluate the dynamic tracking accuracy of ASOFxTSM, a sinusoidal signal is designed for testing purposes. In Case 2, a sinusoidal increasing signal was used, i.e., *φ* = 0.05sin(60*πt*) + 6sin(*πt*) + 5*t* + 9. The numerical simulation results are depicted in Figure 4.

In Figure 4a, the proposed ASOFxTSM is shown to effectively overcome nonlinear factors and successfully track dynamic signals. Compared with FxTSM, ASOFxTSM demonstrates higher tracking accuracy. The MAE values show that ASOFxTSM achieved 6.367 × 10^−3^, FxTSM achieved 28.483 × 10^−3^, and super-twisting achieved 7.173 × 10^−3^. Similarly, the performance of the super-twisting controller on max*E* and MSE will be comparatively worse due to its weaker robustness. 

As evident from Figure 4c, ASOFxTSM effectively maintained stability within the desired area *V*_2−*f*_, leading to a relatively continuous variation in *K*_3_. This characteristic was also evident in the smaller CoEf value of ASOFxTSM compared to the other controllers. For ease of comparison, the data for Case 2 are listed in Table 4.

#### 4.2.3. Trajectory Tracking of Sawtooth (Case 3)

**Figure 5:**
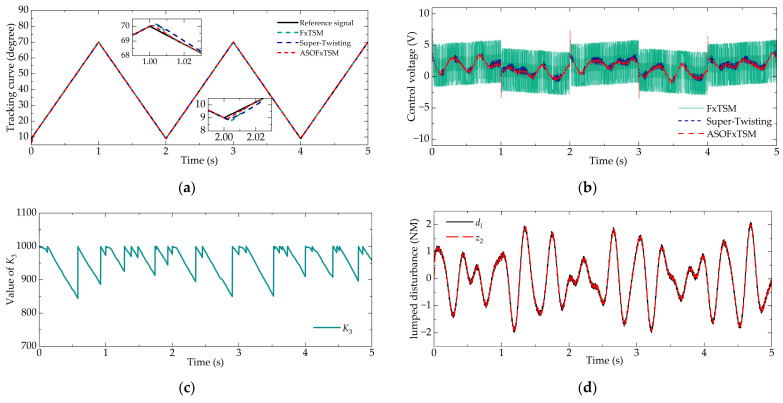
Tracking Performance in Case 3: (**a**) tracking curve, (**b**) control voltage, (**c**) value of *K*_3_, and (**d**) disturbance estimation.

In Case 3, a sawtooth signal was used. This signal is utilized to assess the dynamic tracking performance of the throttle valve during engine acceleration or deceleration. The numerical simulation results are illustrated in Figure 5 and the steady-state criteria are listed in Table 5.

It is evident from Figure 5a that ASOFxTSM exhibited the fastest tracking speed when the signal is switched, and the steady-state error was also minimal. In Figure 5b, ASOFxTSM continues to display excellent output characteristics, providing sufficient output for rapid response. 

To conclude, ASOFxTSM demonstrates exceptional performance and robust stability across the three mentioned working conditions. When compared to conventional control approaches, ASOFxTSM effectively enhances ETV performance while mitigating chattering.

### 4.3. Experiment Setup and Verification

**Figure 6:**
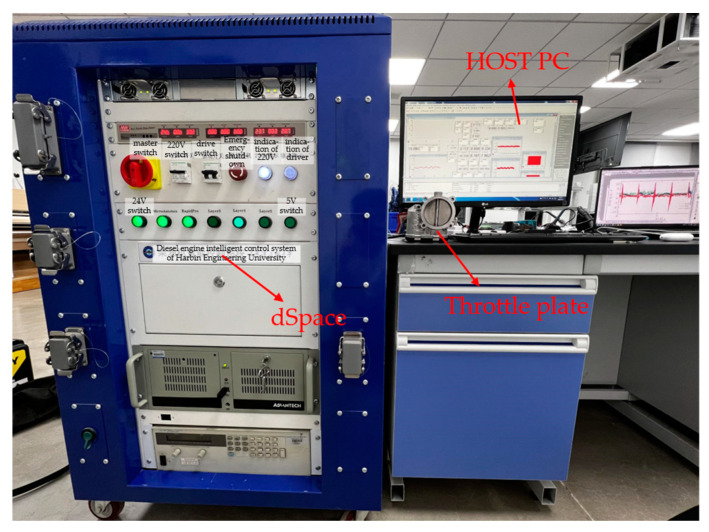
The ETV experimental platform based on RCP.

Experiments are conducted using Rapid Control Prototyping technology and dSpace hardware, as illustrated in Figure 6. The primary objective of these experiments is to validate the advantages demonstrated by the proposed controller in simulation. There are many uncertain factors in the experiment; in order to ensure the stability of the system, the parameter setting is more conservative than the simulation. The parameters of ASOFxTSM are shown in Table 6, the parameters of FxTSM adopts *γ*_1_ = 7/5, *γ*_2_ = 5/9, *α*_1_ = 15, *α*_2_ = 20, *α*_3_ = 10, b_1_ = 7/5, b_1_ = 5/9, *a*_1_ = 5, *a*_2_ = 10, *a*_3_ = 20, *K*_1_ = 500, and the parameters of super-twisting adopts *c*_0_ = 20,*λ*_1_ = 200, *λ*_2_ = 3000.

**Figure 7:**
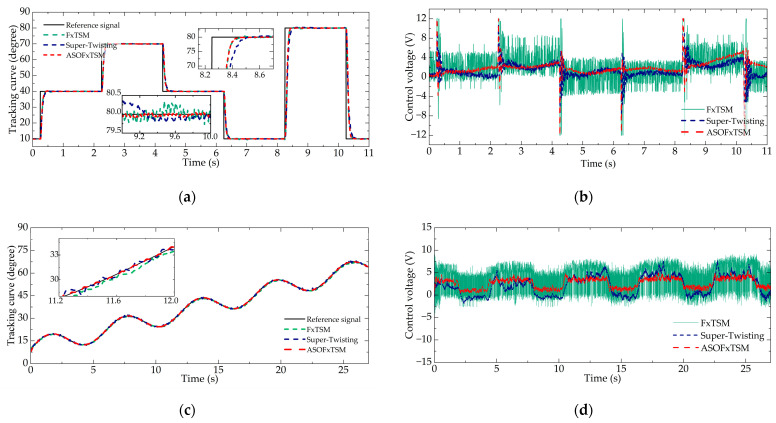

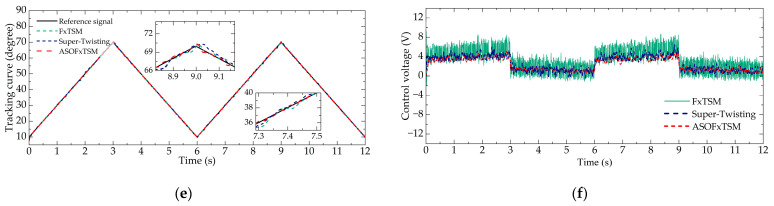
Tracking performance: (**a**) tracking curve in Case 1, (**b**) control voltage in Case 1, (**c**) tracking curve in Case 2, (**d**) control voltage in Case 2, (**e**) tracking curve in Case 3, and (**f**) control voltage in Case 3.

Similar to the simulation, three different reference signals are designed. Figure 7 demonstrates the tracking performance of controllers under these various signals and the corresponding output of controllers.

Figure 7a demonstrates that ASOFxTSM exhibited an excellent performance in the practical application of ETV. For instance, when considering the step signal from 10 to 80 deg, the settling time of ASOFxTSM was 165 ms, while FxTSM required 173 ms, and super-twisting took 235 ms. Additionally, Figure 7c,e display the favorable dynamic tracking performance of ASOFxTSM, with the steady-state error being maintained within ±0.3 deg, whereas FxTSM exhibits ±0.95 deg and super-twisting shows ±0.7 deg steady-state errors. Moreover, Figure 7b,d,f reveal that although the control outputs of ASOFxTSM, FxTSM, and super-twisting varied due to parameter uncertainties and disturbances, ASOFxTSM maintained the smoothest output among them. The experimental performance was evaluated using the above-mentioned criteria, and the steady-state performance of the three working conditions was analyzed to comprehensively evaluate the performance of the controllers. The results are presented in Table 7.

In summary, the proposed controller accomplished accurate tracking control of the ETV despite parameter uncertainties and diverse disturbances. It maintained stability across various operating conditions and exhibits robust resistance to interference, along with minimal chattering, rendering it a more practical solution.

## 5. Conclusions and Discussion

The adaptive second-order fixed-time sliding mode (ASOFxTSM) controller is proposed for the ETV system in this paper, aimed at enhancing the system’s response speed and control accuracy. ASOFxTSM combines fixed-time sliding mode and high-order sliding mode advantages, achieving rapid convergence and effective chattering suppression. The addition of a fixed-time sliding mode observer overcomes overestimation issues caused by difficulty in determining disturbance information. Based on this observer, an adaptive mechanism was added to further reduce chattering. The simulation and experimental outcomes demonstrated that ASOFxTSM attains equivalent convergence speed to FxTSM, far surpassing the asymptotically convergent super-twisting controller. Evaluating diverse metrics reveals ASOFxTSM’s superiority over the other two controllers in terms of steady-state error and controller output. To sum up, the proposed controller adeptly amalgamates the benefits of fixed-time sliding mode and high-order sliding mode, thereby significantly enhancing the effectiveness of electronic throttle valve control.

Furthermore, the controller’s design process involves several parameters, which poses a limitation to this study. As control theory evolves, exploring simpler methods to achieve similar or improved control effects remains a direction for our future research. Simultaneously, we intend to incorporate intelligent algorithms to optimize parameters.

## Figures and Tables

**Table 1:** Electronic throttle valve system parameters.

Parameters	Unit	Nominal Values
*B_m_*	N·m·s/rad	2.4 × 10^−4^
*K_t_*	N·m/A	6 × 10^−3^
*K_m_*	N·m·s/rad	5.6 × 10^−2^
*K_k_*	N·m	2.4 × 10^−4^
*K_e_*	V·s/rad	5 × 10^−6^
*K_s_*	N·m·s/rad	1.5 × 10^−2^
*R*	Ω	2.8
*L*	H	9 × 10^−4^
*J_m_*	kg·m^2^	9 × 10^−4^
*J_et_*	kg·m^2^	8 × 10^−3^
*θ*_0_	rad	1.13 × 10^−1^
*N*	-	16

**Table 2:** Controller parameters for the simulation.

Parameters	Nominal Values	Parameters	Nominal Values
*α*_1_, *α*_2_, *α*_3_	10, 40, 10	*k*_1_, *k*_2_, *k*_3_	100, 200, 100
*γ*_1_, *γ*_2_	7/5, 5/9	*η*_1_, *η*_2_	7/5, 5/9
*β*_1_, *β*_2_, *β*_3_	5, 50, 5	*K_3_*	1000
*ε*_1_, *ε*_2_	7/5, 5/9	*q*	100

**Table 3:** Multi-criteria evaluation of the controllers’ behavior in Case 1.

Controller	MSE (10^−5^)	MAE (10^−3^)	max*E* (10^−2^)	CoEf
FxTSM	115.981	28.497	10.483	1.5406
super-twisting	14.179	5.438	6.259	0.5746
ASOFxTSM	1.117	2.825	1.027	0.5381

**Table 4:** Multi-criteria evaluation of the controllers’ behavior in Case 2.

Controller	MSE (10^−5^)	MAE (10^−3^)	max*E* (10^−2^)	CoEf
FxTSM	114.085	28.483	10.846	1.4096
super-twisting	17.912	7.173	8.043	0.5550
ASOFxTSM	7.182	6.367	1.701	0.5215

**Table 5:** Multi-criteria evaluation of the controllers’ behavior in Case 3.

Controller	MSE (10^−5^)	MAE (10^−3^)	max*E* (10^−2^)	CoEf
FxTSM	111.866	27.957	10.44	1.5502
super-twisting	20.830	6.162	6.260	0.5830
ASOFxTSM	15.822	5.336	0.925	0.5468

**Table 6:** Control parameters for the experiment.

Parameters	Nominal Values	Parameters	Nominal Values
*α*_1_, *α*_2_, *α*_3_	5, 15, 5	*k*_1_, *k*_2_, *k*_3_	50, 300, 80
*γ*_1_, *γ*_2_	7/5, 5/9	*η*_1_, *η*_2_	7/5, 5/9
*β*_1_, *β*_2_, *β*_3_	5, 20, 5	*K_3_*	2000
*ε*_1_, *ε*_2_	7/5, 5/9	*q*	50

**Table 7:** Multi-criteria evaluation of the controller’s overall performance.

Controller	MSE (10^−2^)	MAE (10^−1^)	max*E*	CoEf
FxTSM	6.609	2.049	0.934	1.811
super-twisting	4.124	1.456	0.669	0.790
ASOFxTSM	0.635	0.624	0.292	0.723

## Data Availability

The data used to support the findings of this study are available from the corresponding author upon request.
